# Expression of Thymidine Phosphorylase in Lymph Nodes Involved with Mycosis Fungoides and Sézary Syndrome

**DOI:** 10.1155/2011/875135

**Published:** 2011-11-14

**Authors:** Xingcao Nie, Rekha Bhat, Essel Dulaimi Al-Saleem, Eric C. Vonderheid, J. Steve Hou

**Affiliations:** ^1^Department of Pathology, Drexel University College of Medicine, 245 N. 15th Street, Mail Stop 435, Philadelphia, PA 19102, USA; ^2^Johns Hopkins Medical Institutes, Baltimore, MD, USA

## Abstract

Thymidine phosphorylase may be overexpressed in both neoplastic cells and tumor stromal cells in a variety of malignancies. Our study explores thymidine phosphorylase expression in lymph nodes (LNs) from patients with mycosis fungoides (MF) or Sézary syndrome (SS). In MF/SS, the LNs may have a pathologic diagnosis of either dermatopathic lymphadenopathy (LN-DL) or involvement by MF/SS (LN-MF). We performed immunohistochemical staining on MF/SS lymph nodes using antibodies to thymidine phosphorylase, CD68, CD21, CD3, and CD4. In both LN-DL and benign nodes, thymidine phosphorylase staining was noted only in macrophages, dendritic cells, and endothelial cells. In LN-MF, thymidine phosphorylase expression was also noted in subsets of intermediate to large neoplastic T cells. Concurrent CD68, CD21, CD3, and CD4 staining supported the above observations. Similar results were noted in the skin and in LN-MF with large cell transformation. Other T-cell lymphomas were also examined (total 7 cases); only enteropathy-type T-cell lymphoma (1 case) showed TP positivity in neoplastic T lymphocytes. We demonstrated that thymidine phosphorylase staining is present in neoplastic T cells in mycosis fungoides. The exact mechanism needs further investigation.

## 1. Introduction

Mycosis fungoides (MF) and Sézary syndrome (SS) are the most common types of cutaneous T-cell lymphomas and represent clonal proliferations of neoplastic CD4^+^ T cells. MF initially develops in an indolent fashion as a skin-confined disease. However, with progressive disease, malignant T cells involve skin-associated lymph nodes (LNs), thereby indicating a higher clinical stage [1]. The presence of circulating neoplastic cells in the peripheral blood is the hallmark of SS. Lymphadenopathy in MF/SS ranges from dermatopathic lymphadenopathy (DL), a reactive pattern associated with various chronic skin diseases, to clearcut involvement by malignant T cells that results in partial or complete effacement of lymph node structure (MF-LN). Smaller numbers of atypical or neoplastic cells may be found in DL, but the prognostic significance is unclear [2]. 

Various mechanisms have been implicated in the complexity of tumorigenesis, including imbalance of subsets of T-regulatory cells and cytokine levels [3–5] and dysregulation of chemokine receptors, including CCR4, CXCR4, CCR10 [6–8], and P16 [9]. However, the mechanism of tumorigenesis has not been well established. Thymidine phosphorylase (TP), also known as platelet-derived endothelial cell growth factor, may be overexpressed in both neoplastic cells and tumor stromal cells in a variety of cancers, including breast [10], colorectal [11], gastric [12], esophageal [13], lung [14], and bladder [15]. TP is thought to promote tumorigenesis by inducing tumor cell growth through both inhibition of the apoptosis pathway [16–19] and tumor angiogenesis via the PI3K-mTOR (phosphoinositide 3-kinase-mammalian target of rapamycin) pathway [20]. In addition, TP expression in neoplastic cells can be used as a biomarker to predict response to chemotherapy and survival in esophageal squamous cell carcinoma, breast cancer, and other cancers [21, 22]. However, few studies regarding the role of TP in hematopoietic malignancy have been reported. We studied TP expression in MF-LN. 

## 2. Materials and Methods

Experimental protocols were approved by the Institutional Review Board of Drexel University College of Medicine. Archived paraffin blocks with LNs from 51 patients with MF and SS (1991–2010) were used to construct a tissue microarray. Thirty-four of 51 MF/SS patients had a pathologic diagnosis of DL and 17 had partially or completely effaced LNs by MF or SS. Immunohistochemical (IHC) staining using antibodies to TP, CD68, CD21, CD3, and CD4 was performed. Nine cases of benign LNs were included as control. Archived unstained skin slides were also used to test TP expression from patients with MF in skin-confined stage (5 cases), MF-LN with large cell transformation (1 case), extranodal NK/T-cell lymphoma (1 case), peripheral T-cell lymphoma (2 cases), enteropathy-type T-cell lymphoma (1 case), angioimmunoblastic T-cell lymphoma (2 cases), and syringotropic cutaneous T-cell lymphoma (1 case). 

For IHC staining, the slides were deparaffinized and rehydrated in a series of alcohol. IHC staining was performed using an automated system (Dako Autostainer, Universal Staining System Autostainer, Carpinteria, Calif). The primary antibodies used in this study were TP (1 : 1000; Novus Biologicals, Littleton, Colo), CD3 (1 : 100; Dako), CD4 (1 : 10; Dako), CD68 (1 : 2000; Dako), and CD21 (1 : 50; Vector, Burlingame, Calif), used for tissue microarray blocks. Markers used for skin slides were CD4 and TP; TP was used as a marker for all other T-cell lymphomas.

## 3. Results and Discussion

**Figure 1:**
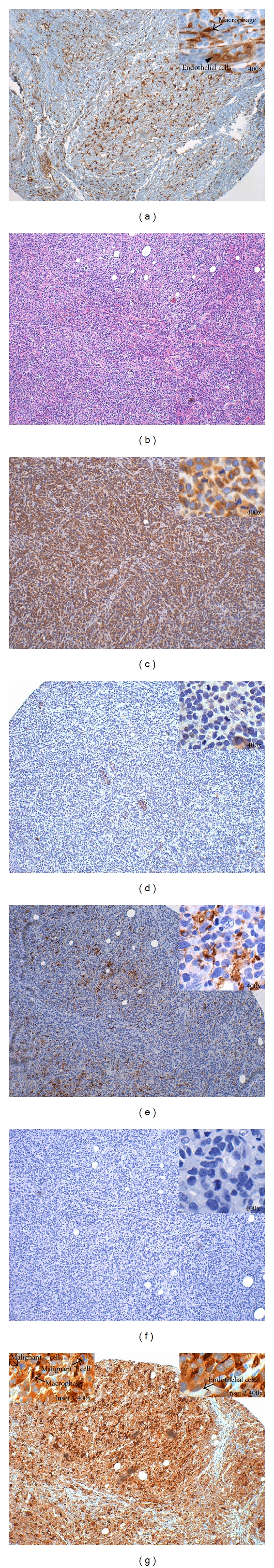
Immunohistochemical staining of thymidine phosphorylase (TP) expression and related markers in benign lymph node (a) and in lymph node with malignant mycosis fungoides/Sézary syndrome cells (LN-MF) (b–g). (a) In benign lymph node, TP expression highlights the meshwork of macrophages (arrow in inset; 400x) and endothelial cells (arrowhead in inset; 400x). (b) LN-MF: hematoxylin and eosin (H&E) stain. (c) CD3 highlights the malignant T cells and reactive T cells in LN-MF. (d) CD4 is weakly expressed in the malignant T cells in LN-MF. (e) CD68 highlights the macrophages in LN-MF. (f) CD21 is negative in this case of LN-MF. (g) TP highlights the macrophages (cytoplasmic/nuclear pattern, arrowhead in inset 1; 400x) and the malignant T cells (cytoplasmic pattern, arrow in inset 2; 400x) in a cytoplasmic staining pattern. Multiple mitotic figures are also noted in the malignant T cells. Images are from an Olympus BX41 microscope (Olympus Corp., Tokyo, Japan) processed with Qcapture Pro 5.1 (QImaging, Surrey, BC, Canada).

**Figure 2:**
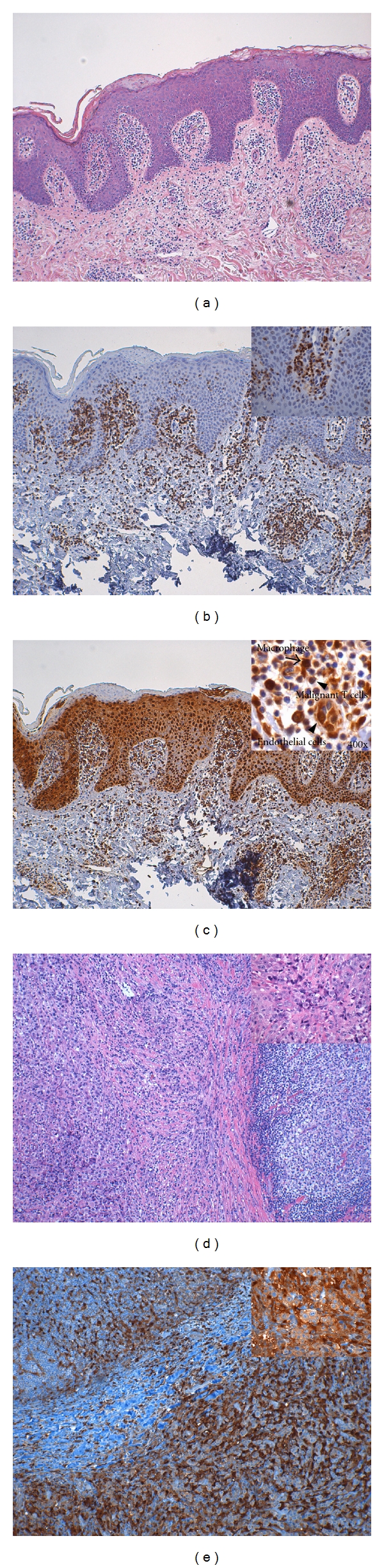
Immunohistochemical stain of thymidine phosphorylase (TP) expression and related markers in skin-involved mycosis fungoides (MF-skin) and in a case of mycosis fungoides/Sézary syndrome cells with involved lymph nodes (LN-MF) with large cell transformation. (a) MF-skin: hematoxylin and eosin (H&E) stain. (b) MF-skin: CD4 highlights the malignant T cells in the epidermis. (c) MF-skin: TP expression is noted in malignant T cells (arrow in inset; 400x), squamous cells, and endothelial cells (arrowhead in inset; 400x). (d) An MF lymph node with large cell transformation is demonstrated: H&E stain. (e) TP stain shows positive immunoactivity in the transformed large neoplastic T cells (arrow in inset; 400x); macrophages are indicated by arrow in inset (400x). Images are from an Olympus BX41 microscope (Olympus Corp., Tokyo, Japan) processed with Qcapture Pro 5.1 (QImaging, Surrey, BC, Canada).

**Figure 3:**
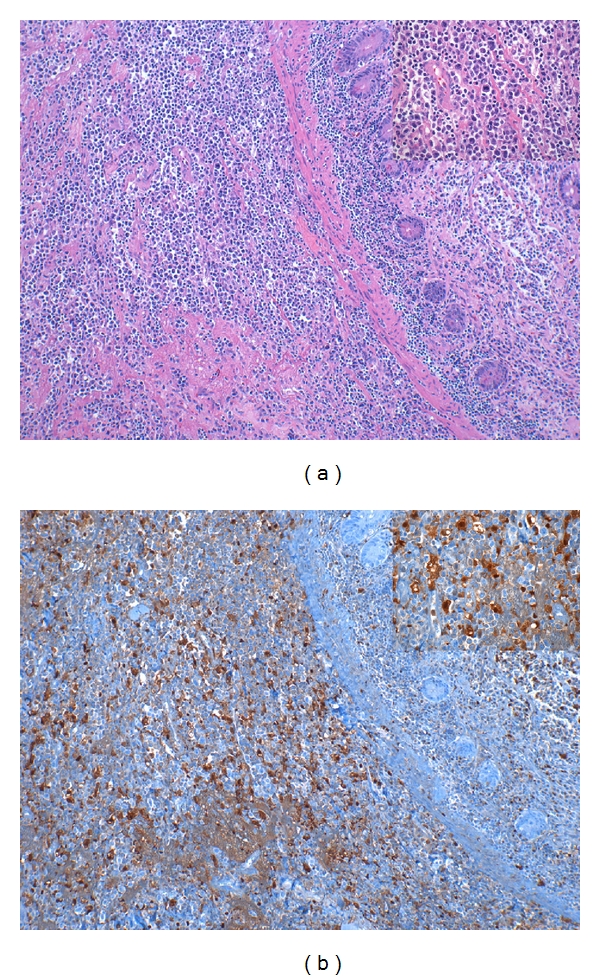
Immunohistochemical stain of thymidine phosphorylase (TP) expression in a gastrointestinal biopsy specimen from a patient with enteropathy-type T-cell lymphoma. (a) Hematoxylin and eosin stain. (b) TP stain highlights neoplastic T cells (arrow in inset; 400x). Images are from an Olympus BX41 microscope (Olympus Corp., Tokyo, Japan) processed with Qcapture Pro 5.1 (QImaging, Surrey, BC, Canada).

In control LNs, TP staining was noted only in macrophages, dendritic cells, and endothelial lining cells, with a cytoplasmic and nuclear staining pattern. Small lymphocytes were negative for TP. A similar TP IHC staining pattern was noted in DL. Positive TP immunostaining was noted in 30% to 80% of intermediate to large neoplastic T cells in all MF-LN cases with a predominantly cytoplasmic staining pattern (Figure 1). Small lymphocytes were negative for TP, and macrophages, dendritic cells, and endothelial lining cells were again positive for TP. Concurrent CD68 and CD21 staining supported the above observations (Figure 1). IHC staining in MF-skin (Figures 2(a)–2(c)) and in MF-LN with large cell transformation (Figure 2(d)) showed a similar staining pattern. Positive TP staining was noted in the neoplastic T cells in one case of enteropathy-type T-cell lymphoma (Figure 3). In other lymphoma cases (extranodal NK/T-cell lymphoma, peripheral T-cell lymphoma, angioimmunoblastic T-cell lymphoma, and syringotropic cutaneous T-cell lymphoma) positive TP staining was only noted in macrophages and endothelial cells. 

Thymidine phosphorylase was found to be present in benign tissue, such as spleen, liver, lymph nodes, esophagus, and rectum, and in a variety of cells, including macrophages, stromal cells, glial cells, endothelial cells, and reticulocytes, with a cytoplasmic or nuclear pattern [23, 24]. Studies have shown that TP is overexpressed in both neoplastic cells and tumor stromal cells in a variety of malignancies such as breast, colorectal, gastric, esophageal, lung, and bladder. We demonstrated that TP expression is present not only in macrophages, dendritic meshworks, and endothelial lining cells but also in intermediate to large neoplastic T lymphocytes.

To our knowledge, 2 previous studies have reported on TP expression in hematopoietic malignancies. In 1990, Yoshimura et al. [24], using immunoblotting, demonstrated higher TP expression in tissues from patients with T- and B-cell malignant lymphomas than from those with lymphoblastic and myeloblastic leukemias. However, they did not determine which cells expressed TP in the hematopoietic malignancies. In 1997 Doussis-Anagnostopoulou et al. [25] used IHC staining to test TP expression in a variety of tissues from patients with hematopoietic malignancies. Malignancies tested included Hodgkin's lymphoma (20 cases), small lymphocytic lymphoma (2 cases), mantle cell lymphoma (1 case), follicular lymphoma (10 cases), diffuse large B-cell lymphoma (8 cases), peripheral T-cell lymphoma (7 cases), intestinal T-cell lymphoma (1 case), and anaplastic large T-cell lymphoma (1 case). TP expression was noted only in the meshwork of dendritic cells, macrophages, and endothelial cells but not in neoplastic cells. The authors commented that the expression of TP in dendritic cells and macrophages might be due to cytokine production and dysregulation contributing to the tumor microenvironment. 

We have demonstrated that TP expression is present in neoplastic T cells in LNs, skin involved with MF or SS (Table 1). This phenomenon may be due to changes intrinsic to the tumor cells themselves, or it may reflect interactions between the tumor microenvironment and the lymphoma cells. The exact mechanism of increased TP expression in lymphoma cells needs further investigation and may have therapeutic implications. Data are scarce regarding TP expression in other neoplastic T-cell lymphomas, and further study would be worthwhile.

## Figures and Tables

**Table 1:** Thymidine phosphorylase immunohistochemical stain (cytoplasmic/nuclear).

	Small lymphocytes	Neoplastic cells	Macrophages and dendritic cells	Endothelial cells
Benign LN	Negative	N/A	Positive	Positive
MF-LN	Negative	Positive	Positive	Positive

LN: lymph nodes; MF-LN: lymph nodes involved with mycosis fungoides or Sézary syndrome.

## Abbreviations

DL: Dermatopathic lymphadenopathy
IHC: Immunohistochemical
LN: Lymph node
MF: Mycosis fungoides
PI3K-mTOR: Phosphoinositide 3-kinase-mammalian target of rapamycin
SS: Sézary syndrome
TP: Thymidine phosphorylase.
